# Investigating the role of Zibai ointment on apoptosis‐related factors Bcl‐2 and Bax in wound healing after anal fistula surgery

**DOI:** 10.1002/iid3.912

**Published:** 2023-06-26

**Authors:** Er‐Wei Cai, Cheng Zhao, Wei‐Juan Wang, Zhen‐Peng Xu, Feng Lin

**Affiliations:** ^1^ The Second Department of Anorectal The Affiliated People's Hospital of Fujian University of Traditional Chinese Medicine Fujian China; ^2^ Paramedics The Affiliated People's Hospital of Fujian University of Traditional Chinese Medicine Fujian China

**Keywords:** anal fistula, Bax, Bcl‐2, Zibai ointment

## Abstract

**Objective:**

In this study, we investigated the impact of Zibai ointment on wound healing by analyzing the expression levels of two key apoptosis‐related factors—B‐cell lymphoma 2 (Bcl‐2) and Bcl‐2‐associated X protein (Bax), in patients following surgery for anal fistula.

**Methods:**

We included 90 patients with anal fistulas who were treated in the People's Hospital Affiliated to Fujian University of Traditional Chinese Medicine. Patients were randomly assigned to receive treatment with Zibai ointment (*n* = 45) or petroleum jelly (*n* = 45). The levels of apoptosis‐related factors Bcl‐2 and Bax were evaluated using enzyme‐linked immunosorbent assay (ELISA), while cell apoptosis was assessed using Terminal deoxynucleotidyl transferase (TdT) dUTP Nick‐End Labeling (TUNEL) assay.

**Results:**

The results of ELISA showed that on Day 21 after the surgery, the levels of Bcl‐2 and Bax in the Zibai ointment group were significantly different compared to the petroleum jelly group, with values of (60.11 ± 1.31) ng/mL and (7.05 ± 0.01) versus (83.79 ± 1.74) ng/mL and (6.00 ± 0.05) ng/mL, respectively (*p* < .05). Furthermore, light microscopy revealed a large number of apoptotic cells within the field of vision 14 days postsurgery in the Zibai ointment group, and the healing time in the Zibai ointment group was significantly different from that in the petroleum jelly group (*p* < .05).

**Conclusion:**

We found that Zibai ointment effectively promoted wound healing in patients following anal fistula surgery, possibly by regulating Bcl‐2 and Bax apoptosis‐related factors.

## Graphical abstract

In this study, we found that Zibai ointment effectively promoted wound healing in patients after anal fistula surgery, possibly by regulating Bcl‐2 and Bax apoptosis‐related factors.

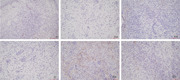

## 1 INTRODUCTION

1.1

Common anorectal diseases like anal fistula can only be treated surgically. However, postoperative wound healing can be challenging and unpleasant due to the anatomical features of the lesion site and the surrounding area. Patients experience a great deal of discomfort due to the lengthy postoperative recovery period. Therefore, it is crucial to find efficient methods to improve wound healing following surgery for anal fistula. Clinical studies have shown that Zibai ointment is highly effective in the postoperative treatment of anal fistula. We designed this preliminary study to explore the potential effects of Zibai ointment on cell apoptosis in promoting wound healing. The details are reported in the following sections.

## 2 MATERIALS AND METHODS

### 2.1 Clinical materials

#### 2.1.1 Diagnostic criteria

The diagnostic criteria for anal fistula used in this study are based on the *Clinical Guidelines for the Diagnosis and Treatment of Anal Fistula*,^1^ jointly published by the Anorectal Society of Chinese Society of Traditional Chinese Medicine (TCM), the Colorectal and Anal Surgery Group of Surgery Society of Chinese Medical Association, and the Professional Committee of Colorectal and Anal Diseases of Chinese Association of Integrated Traditional Chinese and Western Medicine. The anal fistula in the study population was a low, simple anal fistula—there was only one fistula, the fistula was located below the depth of the external sphincter, and the fistula mouth was near the anal sinus. The main clinical symptoms of downward flow of damp‐heat in anal fistula, according to the *Guidelines for the Diagnosis and Treatment of Common Diseases in Anorectal Medicine of* *TCM*,^2^ include perianal ulceration, frequent pyorrhea with thick white or yellow pus, local redness and swelling, heat and pain, cord‐like substance leading to the anus when pressed, accompanied by indigestion, loss of appetite, poor bowel movements, scanty dark urine, heavy limbs, a red tongue, yellow and greasy coating on the tongue, and rapid and slippery pulse.

#### 2.1.2 Inclusion criteria

(1) Patients who met the above diagnostic criteria for anal fistula; (2) Patient aged > 18 years and <60 years; (3) The surgical method used was conventional anal fistula resection; (4) All surgeries were performed under either local infiltration anesthesia or lumbar anesthesia; (5) Patients without previous history of anal fistula surgery, or abnormal anal morphology and function; (6) Patients who agreed to be treated with Zibai ointment and gave their signed informed consent.

#### 2.1.3 Exclusion criteria

(1) Patients with other anal diseases, such as anal fissure, perianal abscess, perianal eczema, rectal polyps, anal papilloma; (2) Patients suffering from chronic diarrhea or other gastrointestinal infections; (3) Patients diagnosed with malignant tumors in the body; (4) Patients with diseases affecting vital organs such as heart, brain, liver, lung, kidney, or diagnosed with psychiatric illnesses; (5) Pregnant and lactating women; (6) Patients allergic to Zibai ointment; (7) Patients who underwent anesthesia and surgical procedures that were different from those for inclusion in this study, or with incomplete data that impacted the efficacy assessment.

#### 2.1.4 General data

The study was approved by Ethics Committee of the People's Hospital Affiliated to Fujian University of TCM. Written informed consent was obtained from all participants. (2020‐048‐02). Patients were randomly assigned to either the Zibai ointment group (*n* = 45) or the petroleum jelly group (*n* = 45) by the researchers. Randomized number table method was used to group the treatment group and control group in a 1:1 ratio, and the “envelope method” was used to hide the random allocation plan. According to the random number table, 90 consecutive random numbers are randomly selected. It is stipulated that if the random number is odd, it will be assigned to the control group, and if it is even, it will be assigned to the treatment group. Place the randomly generated allocation order into a coded, sealed, and opaque envelope. After the researcher determines that the subject meets the eligibility criteria for inclusion, open the envelope in the order of hospitalization surgery and place the subject information in the corresponding experimental group. There was no statistical significance in gender and age differences between the groups (*p* > .05) and the groups were well‐matched (Table 1).

**Table 1:** Comparison of Gender and Age between the Two Groups.

Group	Gender		Age ($\bar{X}\;\pm$ S) years
	Male	Female	
Zibai ointment group	31	14	37.42 ± 10.43
Vaseline group	25	20	36.62 ± 10.09
	*χ* ^2^ = 0.277		*F* = 0.291
	*p* = .138		*p* = .591

### 2.2 Methods

#### 2.2.1 Treatment methods

Surgical method: First, use a probe to gently probe along the direction of the fistula from the outer opening, pass through the entire fistula, and reach the inner opening. Cut all fistulas along the direction of the probe and open the entire length of the fistula. Make incisions on both sides of the skin of the open fistula, continue to deepen along the incision, and cut diagonally towards the deep part of the fistula to remove the entire fistula. The transverse section of the wound is V‐shaped. Patients in both groups received standard postoperative anorectal care, and the wound dressing was routinely changed on the second day after surgery. Patients were asked to avoid eating spicy and fried foods during treatment and maintain regular bowel movements. The Zibai ointment group received external application of Zibai ointment (People's Hospital Affiliated to Fujian University of TCM, MYZZ Z06106031) on the first day postsurgery, while the control group received external application of petroleum jelly (production license No.: Zhejiang Food and Drug Administration Equipment Production License No. 20100048). The wound healing time was recorded, and we compared the clinical efficacy between the groups.

#### 2.2.2 Experimental methods

We collected granulation tissue samples from both groups on Days 7, 14, and 21 postsurgery to analyze Bcl‐2 and Bax levels using enzyme‐linked immunosorbent assay (ELISA) and to measure granulation tissue apoptosis using the TUNEL assay.

We used Human B lymphocytoma‐2 (Bcl‐2) ELISA kit (enzyme immunoassay, MM‐0381H1) B lymphocytoma‐2 associated X protein (Bax) ELISA kit (enzyme immunoassay, MM‐1143H1), and the TUNEL cell apoptosis kit (Beyotime, C1098).

##### ELISA method for detecting the expression of Bcl‐2 and Bax

An appropriate amount of tissue was collected for later use after removal of blood, and weighed and transferred into a glass homogenizer. The tissue was rinsed with 5–10 mL of precooled PBS (0.01 M, pH = 7.4) to eliminate any residual blood and then ground thoroughly. The prepared homogenate was centrifuged at 5000*g* for 5 min, and the supernatant was saved for analysis. We followed the specific procedures as per the ELISA kit instructions.

##### TUNEL assay for assessing cell apoptosis in granulation tissue postsurgery

The granulation tissue was prepared for apoptosis detection by fixing it, embedding in paraffin, and sectioning and dewaxing with xylene. It was then digested with trypsin, incubated with the TUNEL reaction solution at 37°C for 1 h, then incubated with peroxidase antibody at 37°C for 30 min; diamine benzidine (DAB) was added dropwise, the sample sections were incubated at room temperature for 30 min, then sealed and dried. The sample sections were then immersed in hematoxylin staining solution for nuclear staining for 5 min. The apoptosis rate was determined under a microscope by counting the cell nuclei stained with brownish‐yellow particles as apoptotic positive cells and the apoptosis rate (%) was calculated—apoptosis rate = (number of positive cells/total number of cells) × 100%.

### 2.3 Statistical analysis

We used SPSS 25.0 software for the data analysis. Measurement data are expressed as mean values and the means of two samples were compared using the *t*‐test. If the requirements for *t*‐test were not met, we used the Wilcoxon rank sum test to compare the means. Enumeration data were compared using the chi‐squared test. A *p* < .05 was considered statistically significant.

## 3 RESULTS

### 3.1 Levels of Bcl‐2 and Bax in the two groups after surgery

The levels of Bcl‐2 gradually decreased and those of Bax gradually increased in both groups over time. On Day 21 post‐surgery, there were significant differences in the expression levels of Bcl‐2 and Bax between the groups (*p* < .05), suggesting that the Zibai ointment regulated the expression of Bcl‐2 and Bax, facilitating the wound healing process (Table 2).

**Table 2:** Expression Levels of Bcl‐2 and Bax in the postoperative tissues of the two groups.

Group	Number of cases	Time	Bcl‐2 (ng/mL)	Bax (ng/mL)
Zibai ointment group	45	1 day	118.90 ± 4.26	2.61 ± 0.02
		7 day	102.39 ± 0.43	3.35 ± 0.01
		14 day	89.28 ± 2.18	5.54 ± 0.11
		21 day	60.11 ± 1.31^#^	7.05 ± 0.01^#^
Vaseline group	45	1 day	125.21 ± 1.31	1.46 ± 0.03
		7 day	108.15 ± 1.98	2.75 ± 0.02
		14 day	92.66 ± 1.81	3.45 ± 0.04
		21 day	83.79 ± 1.74^#^	6.00 ± 0.05^#^
	*F*		54.800	495.628
	*P*		.000	.000

^#^ The expression of Bcl‐2 in Zibaigao group was compared with that in petroleum jelly on the 21st day after operation, *F* = 54.800, *P* = .000; Compared with the petroleum jelly, the expression of Bax was *F* = 495.628, *P* = .000.

### 3.2 TUNEL assay results on cell apoptosis in wound granulation tissue

**Figure 1:**
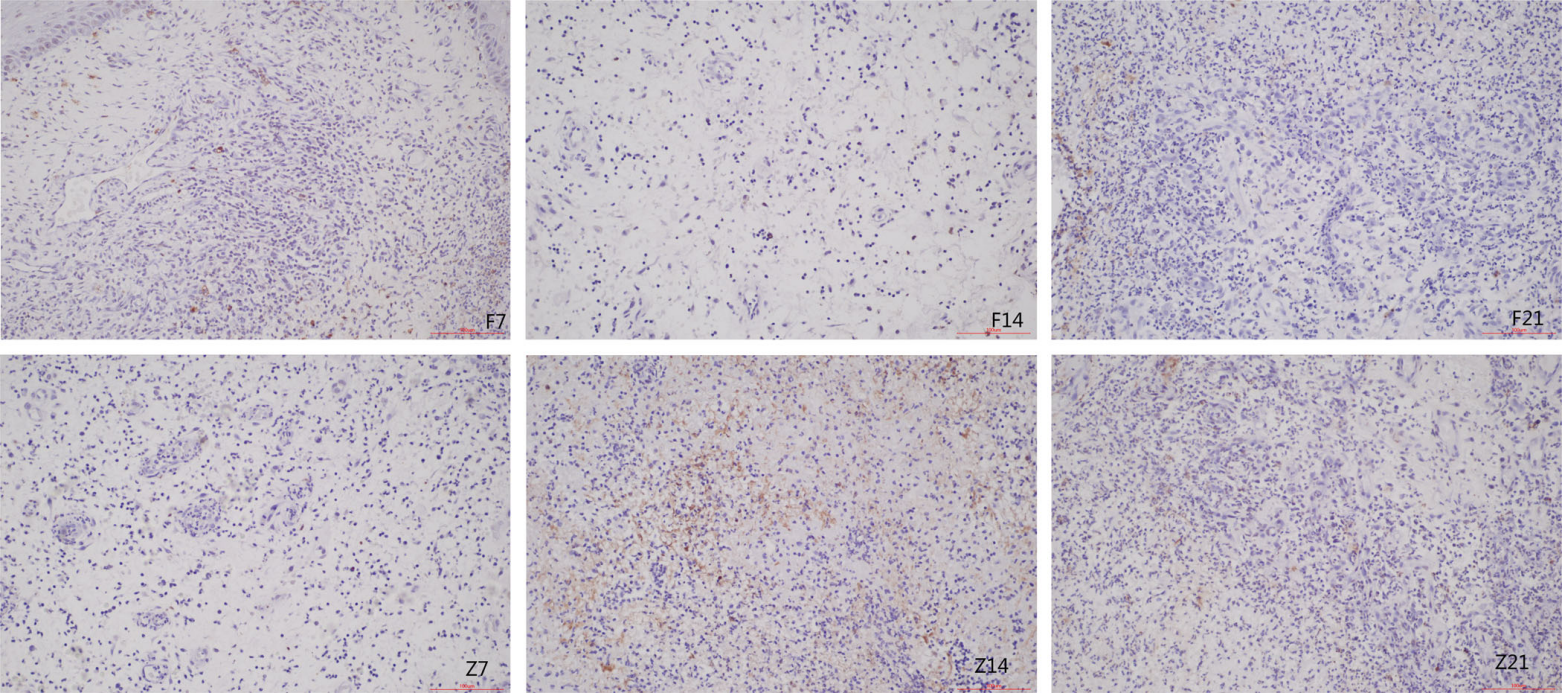
Apoptosis in each group detected by TUNEL assay (×200).

On Day 14 post‐surgery, samples from the Zibai ointment group showed signs of apoptosis (brown‐yellow cells with nuclei) under light microscopy, including chromatin condensation (arrangement close to the karyotheca in a hemisphere‐shaped, crescent‐shaped or sickle‐shaped manner), cytoplasmic pyknosis, reduced cell volume, loose cell arrangement, irregular‐shaped cells, and occasional apoptotic bodies. The apoptotic cells in the petroleum jelly group decreased significantly at the same time point. We did not find any significant differences in the proportion of positive cells between the Zibai ointment and petroleum jelly control groups on Days 7 and 21 postsurgery (*p* > .05), however, there was a significant difference on Day 14 post‐surgery (*p* < .05), and the apoptosis index (AI) of the two groups showed an incremental relationship (Figure 1 and Table 3).

**Table 3:** Apoptosis index of in situ end‐labeling ($\bar{x}\;\pm s$).

Group	Number of cases	Time	Total number of cells (cells)	Number of positive cells (cells)	AI%
Zibai ointment group	45	7 day	548	103^a^	18.11 ± 3.24
		14 day	625	110^b^	18.62 ± 14.89
		21 day	508	98^c^	24.10 ± 16.31
Vaseline group	45	7 day	660	106^a^	15.24 ± 16.54
		14 day	547	66^b^	11.60 ± 4.64
		21 day	546	121^c^	24.16 ± 7.85

^a^ The number of apoptotic positive cells in the Zibai ointment group on Day 7 after operation, compared with the Vaseline group, *χ* ^2^ = 1.123, *p* = .289;

^b^ The number of apoptotic positive cells in the Zibai ointment group on Day 14 after operation, compared with the Vaseline group, *χ* ^2^ = 4.823, *p* = .028;

^c^ The number of apoptotic positive cells in the Zibai ointment group on Day 21 after operation, compared with the Vaseline group, *χ* ^2^ = 0.847, *p* = .357.

### 3.3 Comparison of postoperative healing time and clinical efficacy between the two groups

There was no significant difference in clinical efficacy between the two groups (*p* > .05) though there was improvement in both groups. However, patients in the Zibai ointment group had a shorter healing time compared to the petroleum jelly group and this difference was statistically significant (*p* < .05) (Table 4).

**Table 4:** Comparison of Postoperative Healing Time and Clinical Efficacy between the Two Groups.

Group	Healing time	Cure rate (number of cases)		
		Complete response	Moderate response	No response
Zibai ointment group	26.49 ± 4.19	43	2	0
Vaseline group	30.38 ± 4.45	40	5	0
	Z = −3.80		*χ* ^2^ = 1.394	
	*p* = .000		*p* = .238	

## 4 DISCUSSION

Anal fistula is an anorectal disorder with high incidence, characterized by the formation of an abnormal tunnel between the rectum or anal canal and surrounding skin. Perianal abscess, pruritus, and pain are some of its clinical signs. Systemic inflammatory reactions, fever, and other symptoms may also be present in more severe cases. Due to the impossibility of self‐healing, surgical intervention is the therapeutic treatment of choice.^3^ However, the difficult‐to‐heal postoperative wound and pain make it a clinical challenge to promote quick and effective healing.^4^ Healing of chronic wounds is essentially a process of repairing chronic inflammation, including alleviation of inflammatory response, dissolution, and granulation. According to new research, apoptosis is not only an integral aspect of the wound healing process, but also the simplest and most fundamental method for decreasing cell numbers.^5^

Apoptotic processes are regulated by antiapoptotic (Bcl‐2) family proteins and Caspase family proteins by regulating the transduction of apoptotic signals, and their mutual checks and balances form a complete and efficient apoptosis mechanism network system.^6^ The apoptotic signaling pathways include the proapoptotic protein Bax and the antiapoptotic protein Bcl‐2.^7^ Bcl‐2 can inhibit cell apoptosis mainly by blocking the signaling pathway of apoptosis, while the expression of Bax can promote cell apoptosis.^8^

In the Bcl‐2 gene family, the apoptosis‐promoting gene Bax has been researched extensively due to its high degree of similarity with Bcl‐2. Bax has the ability to suppress Bcl‐2 by forming homodimers or heterodimers with Bcl‐2. Consequently, the antiapoptotic proteins Bcl‐2 and Bax are essential for regulating cell apoptosis.^9^ The antiapoptotic Bcl‐2 is a member of the Bcl‐2 family and a major target gene of the apoptotic mechanism. When Bcl‐2 is silenced, Bax dissociates from the heterodimer of Bcl‐2, resulting in an increase in the expression of Bax. At the same time, the Bcl‐2 homodimer is also reduced. Ultimately, this activates caspase‐3, thus promoting cell apoptosis.^10^ Therefore, the balance of the proportion between the two is a key factor to determine the strength of the inhibitory effect on apoptosis.^11^

According to TCM, anorectal diseases are caused by stagnation of qi and blood and the downward flow of damp‐heat. Many TCM remedies aim to activate blood, remove stasis, alleviate pain, clear heat, dryness, and dampness, and reduce swelling. External therapies with these TCM remedies are widely used for the treatment of postoperative pain and edema complications in anorectal disorders due to their direct effect, minimal toxicity and side‐effects, safety, and convenience.

Research has demonstrated the effectiveness of TCM in promoting wound healing, reducing pain, and suppressing the inflammatory response in patients after anal fistula surgery.^12^, ^13^, ^14^ Zibai ointment is a medicinal extract for external application that is extensively used to treat patients in the anorectal department of our hospital. It has proven clinical efficacy in treating postoperative complications of anorectal diseases. The ointment contains a blend of Chinese herbs that work together to clear heat and dryness, activate blood circulation, dissipate blood stasis, remove necrotic tissue, promote granulation, heal sores, and facilitate wound healing. The key ingredients are *Rheum officinale*, which can clear away heat, purge pathogenic fire, promote purgation, remove food retention, stop bleeding, clear toxic matter, promote blood circulation, and remove stasis, and *Arnebiae radix*, which can remove pathogenic heat from blood and promote blood circulation, remove heat and toxic matter. Along with rhubarb, *Arnebiae radix* and *R. officinale* constitute the monarch drugs to enhance the effects of clearing away heat and toxic materials, promote blood circulation, dissipate blood stasis, and relieve pain. Additionally, gypsum can clear away heat and purge pathogenic fire, heal sores, and promote granulation while *Rhizoma bletillae* can stop bleeding via astringency, relieve swelling, and promote granulation. These two constitute the ministerial drugs to strengthen the heat‐clearing and fire‐purging effect of monarch drugs and enhance the effect of removing necrotic tissue and promoting granulation, healing sores, and promoting granulation, thus facilitating wound healing.

Borneol, used as an adjuvant drug, clears heat, reduces swelling, relieves pain, and eliminates stagnation. According to TCM principles, the combination of these herbs in Zibai ointment helps to clear heat, dries out dampness, invigorates blood circulation, eliminates stasis, removes necrotic tissue, promotes granulation, heals sores, and fosters healing of wounds.

The results of our study revealed statistically significant differences in the expression levels of Bcl‐2 and Bax between the Zibai ointment and petroleum jelly groups on Day 21 postsurgery (*p* < .05), suggesting that Zibai ointment induced the increase of Bcl‐2/Bax ratio during wound healing, and this may be one of the important targets of its action. In the TUNEL assay, we found several apoptotic cells in the field of vision in the Zibai ointment group on Day 14 postsurgery, indicating its potential to effectively promote cell apoptosis and improve wound healing at the peak period of granulation growth 2 weeks after surgery, thus improving wound metabolism and shortening the wound healing time. The healing time in patients in the Zibai ointment group was shorter than that in the petroleum jelly group and the difference between the groups was statistically significant (*p* < .05). Overall, we found that Zibai ointment was effective in promoting wound healing and shortening the healing time after anal fistula surgery.

There is some preliminary evidence that Zibai ointment can promote wound healing following anal fistula surgery by controlling cell apoptosis. However, Zibai ointment induces an increase in the Bcl‐2/Bax ratio by regulating upstream factors of the Bcl‐2/Bax signaling pathway. Wound healing is aided by the ability of Zibai ointment to directly regulate the expression of Bcl‐2 and Bax, thereby promoting cell apoptosis during wound healing. All these issues need to be explored in greater detail. Using either multicenter studies or animal models, the study team can delve more deeply into the underlying mechanism of Zibai ointment in future investigations, providing a more objective basis for its clinical application.

Study limitation: Due to uncertain factors such as the time limit of the study and patient compliance, the clinical sample size of this study is relatively small, but some significant differences have also been initially found. In subsequent studies, the research team can conduct multicenter studies or use animal models to deeply explore the internal mechanism of Zibai ointment, providing a more objective basis for its clinical application.

## AUTHOR CONTRIBUTIONS

**Er‐Wei Cai**: Conceptualization; funding acquisition. **Wei‐Juan Wang**: Formal analysis; funding acquisition. **Zhen‐Peng Xu**: Funding acquisition. **Feng Lin**: Formal analysis; funding acquisition.

## CONFLICT OF INTEREST STATEMENT

The authors declare no conflict of interest.

## ETHICS STATEMENT

The study was conducted in accordance with the Declaration of Helsinki(as was revised in 2013). The study was approved by Ethics Committee of the People's Hospital Affiliated to Fujian University of Traditional Chinese Medicine. Written informed consent was obtained from all participants (2020‐048‐02).
